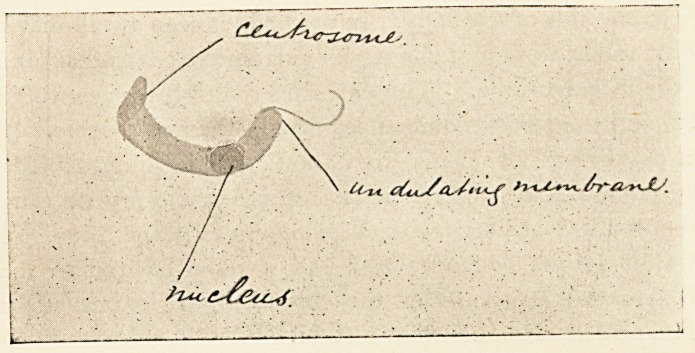# Trypanosomiasis

**Published:** 1903-12

**Authors:** J. Odery Symes

**Affiliations:** Assistant Physician and Bacteriologist, Bristol General Hospital.


### TRYPANOSOMIASIS.

BY

J. Odery Symes, M.D., D.P.H. Lond.

*Assistant Physician and Bacteriologist, Bristol General Hospital.*

---

The blood-films which I have shown[1] were taken from a case of Dr. Skerritt's which was under my care for a period of two months during the past summer. The clinical history of the case will be found in the *British Medical Journal*, May 30th, 1903. The patient died on November 26th, and details of the *post-mortem* examination will be published shortly.

Trypanosomes belong to the order of Protozoa. They have been known as the cause of certain pathological conditions in animals, viz.: the disease known as "surra" amongst cattle in India; "nagana" (tse-tse fly disease) in S. Africa, S. America and Mauritius; "dourine" (a form of equine gonorrhœa) in N. Africa and S. Europe; "caderas," a disease of horses in S. America; and an unnamed newly discovered cattle disease in the Transvaal. Many varieties of rats are, too, infested with trypanosomes, and the parasites may be found to be almost as numerous in the blood as the red blood corpuscles.

[1] Shown at the Meeting of the Society, November 11th, 1903.

The parasites are communicated to the animals by the bites of fleas or flies.

It is not known that the trypanosomes of cattle are identical with those found in human blood or can be transmitted to man.

Human trypanosomes have been successfully inoculated on monkeys, producing the symptoms of typical sleeping sickness.

*Trypanosomes in cerebro-spinal fluid.*

Trypanosomiasis in man was first fully described by Dutton in 1902. The clinical features of the disease are: undulant, intermittent fever of prolonged duration not yielding to quinine, erythema, œdema, muscular weakness, rapid irritable pulse, anæmia, breathlessness, enlargement of the spleen, mononuclear leucocytosis, choroiditis, and iritis.

The parasite is best found in the blood at the height of the pyrexia but may be absent for days together. At any time it is difficult to detect, as not more than five or six parasites are generally present in a blood-film spread on a 3-inch slide. The trypanosome in a fresh hanging-drop preparation of blood is seen swimming freely in the blood plasma. It is a transparent vermicule, having at its posterior end a long flagellum which is connected with an undulating membrane which is fixed like a flange to one side of the body. In the body is seen a well-marked nucleus, and at the blunted anterior extremity is a bright shining spot, the "centrosome." The parasites multiply by longitudinal fission, and the fission commences first at the centrosome.

In preparing specimens from the blood the films should be spread fairly thickly and stained as soon as dry. Trypanosomes can be readily stained with Löffler's methylene blue, with thionin, or Jenner's blood stain; but if it be desired to show the differential structure of the organism, it is best to use Leishmann's or Romanowsky's stain.

As human trypanosomes measure from 18—25$\mu$ in length, more than three times as long as a red blood cell, by 2—3$\mu$ in width, they are readily detected by a $\frac{1}{6}$-inch objective.

We do not yet know how trypanosomes are communicated to man, but probably it is by the bite of the Uganda tse-tse fly (Glossina palpalis). Another point not yet cleared up is as to whether there be any causal relationship between trypanosomiasis and sleeping sickness. The arguments in favour of this relationship are as follows: The trypanosome is constantly present in the cerebro-spinal fluid of persons suffering from sleeping sickness, and when inoculated into monkeys produces the symptoms of this disease. It is not found in the cerebro-spinal fluid of other diseases. The pathological changes of sleeping sickness and the mononuclear blood-count resemble those of trypanosomiasis. The fly which is supposed to act as a host to the human trypanosome is only found in the districts where sleeping sickness is endemic. On the other hand, recent investigations have shown that trypanosomiasis is a fairly common condition amongst natives in certain districts, and may give rise to few or no symptoms, so that the association of sleeping sickness and infection by trypanosomes may be accidental. Not more than three or four cases of trypanosomiasis in white persons have been recorded, and none of these have developed symptoms of sleeping sickness.

The presence of a streptococcus in the blood of persons suffering from sleeping sickness is regarded by Castellani as a concomitant infection.

## Figures and Tables

**Figure f1:**
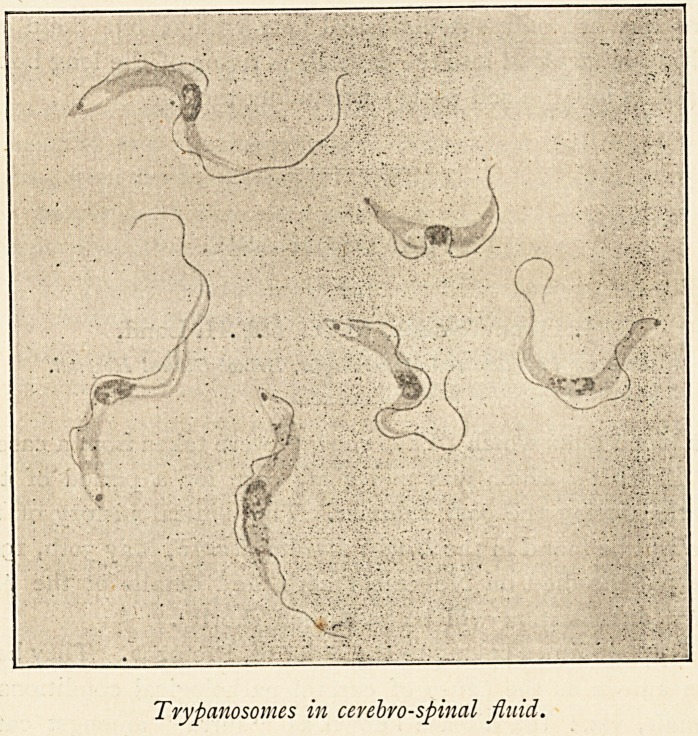


**Figure f2:**